# Resveratrol-Loaded Levan Nanoparticles Produced by Electrohydrodynamic Atomization Technique

**DOI:** 10.3390/nano11102582

**Published:** 2021-09-30

**Authors:** Ezgi Cinan, Sumeyye Cesur, Merve Erginer Haskoylu, Oguzhan Gunduz, Ebru Toksoy Oner

**Affiliations:** 1 Industrial Biotechnology and System Biology (IBSB) Research Group, Department of Bioengineering, Marmara University, Istanbul 34722, Turkey; ezgicinan35@gmail.com (E.C.); merweerginer@hotmail.com (M.E.H.); 2 Center for Nanotechnology & Biomaterials Application and Research (NBUAM), Marmara University, Istanbul 34722, Turkey; sumeyye-cesur@hotmail.com (S.C.); ucemogu@ucl.ac.uk (O.G.); 3 Department of Metallurgical and Materials Engineering, Faculty of Technology, Marmara University, Istanbul 34722, Turkey

**Keywords:** *Halomonas* levan, polymeric nanoparticles, resveratrol, electrohydrodynamic atomization, drug delivery systems

## Abstract

Considering the significant advances in nanostructured systems in various biomedical applications and the escalating need for levan-based nanoparticles as delivery systems, this study aimed to fabricate levan nanoparticles by the electrohydrodynamic atomization (EHDA) technique. The hydrolyzed derivative of levan polysaccharide from *Halomonas smyrnensis* halophilic bacteria, hydrolyzed *Halomonas* levan (hHL), was used. Nanoparticles were obtained by optimizing the EHDA parameters and then they were characterized in terms of morphology, molecular interactions, drug release and cell culture studies. The optimized hHL and resveratrol (RS)-loaded hHL nanoparticles were monodisperse and had smooth surfaces. The particle diameter size of hHL nanoparticles was 82.06 ± 15.33 nm. Additionally, release of RS from the fabricated hHL nanoparticles at different pH conditions were found to follow the first-order release model and hHL with higher RS loading showed a more gradual release. In vitro biocompatibility assay with human dermal fibroblast cell lines was performed and cell behavior on coated surfaces was observed. Nanoparticles were found to be safe for healthy cells. Consequently, the fabricated hHL-based nanoparticle system may have potential use in drug delivery systems for wound healing and tissue engineering applications and surfaces could be coated with these electrosprayed particles to improve cellular interaction.

## 1. Introduction

Recent advancements in biotechnology and medicine allow nanoparticles to be used intensively in biomedical applications, including production and improvement of delivery systems for bioactive molecules or chemotherapeutic agents, diagnosis and therapy of a variety of diseases and tissue engineering [1,2,3,4,5,6]. Nanoparticles fabricated for drug delivery reduce their side effects, while enhancing drug efficiency and improving drug release and pharmacological properties [7]. Various materials including polymers, metals, ceramics, lipids, and carbon nanotubes have been used to produce these nano-sized carriers. Among these, polymer-based nanoparticles are versatile carriers with many crucial characteristics like stability and allowing a high load of agents, easy-to-modify, and proving control of drug release kinetics [8]. There is a continuing interest and challenge in biocompatible and biodegradable nanoparticle manufacturing from polymers in the biomedical industry [9,10,11]. Polymeric nanoparticles are colloidal particles that can be produced by using natural or synthetic polymers [2]. Among the polymers, the most recognized biopolymers are polysaccharides which are naturally derived compounds as chains of carbohydrate molecules [12,13]. Polysaccharide-based polymers have received significant interest for their use in the fabrication of a drug delivery system as carriers due to their outstanding biocompatibility, biodegradability, ability to promote the release of drug/therapeutic agent, low toxicity, mostly less immunogenic activity, cost-efficiency and stability [1,2,14,15]. There are various polysaccharides such as alginate, xanthan gum, starch, curdlan, chitosan, levan, dextran and hyaluronic acid that are used in drug delivery systems as carriers [13].

Levan is a fructan-type naturally occurring polysaccharide composed mainly of β-(2→6) linked β-d-fructofuranose residues [16,17,18]. Levan can be in the form of linear and also branched by the contribution of β-(2→1) linkages [16,17,19]. It is produced mainly from microorganisms as well as a few plants. Halophilic *Halomonas smyrnensis* cultures were represented as the first extremophilic levan producers providing high yields and cost-effective production under non-sterile and highly saline conditions [20,21,22]. Levan is a unique polymer with properties such as biocompatibility, notably low intrinsic viscosity, and higher stability due to its structural arrangement and high adhesivity [19,23]. Our previous studies on levan produced by *H. smyrnensis* cultures, *Halomonas* levan (HL), and its derivatives have shown that they are versatile biopolymers with anticancer [24], heparin-mimetic anticoagulant activities [25] forming bioactive thin film blends and adhesive multilayer films [26,27] or carrier systems for antibiotics, drugs and proteins [11,28] that can be used for a wide range of applications such as nano/micro drug delivery systems, tissue engineering or regenerative medicine. Additionally, HL is known to form self-assembled spheroids in water due to its high molecular weight and intensive hydrogen bonding [16]. Moreover, Avsar et al. (2018) showed that reducing the molecular weight by hydrolysis also increased the levan solubility and hydrolyzed (hHL) and sulphated (ShHL) HL based fibrous scaffolds were successfully prepared via single-needle and co-axial electrospinning [29].

Resveratrol (RS) (trans-resveratrol; (trans-3,4′,5,-trihydroxystilbene) is a polyphenolic molecule from the stilbene family, and grapes skin and seeds, the roots of *Polygonum cuspidatum,* pomegranate, and nuts are examples of its naturally available sources. As a biologically active molecule displaying anti-cancer, antioxidant, anti-inflammatory, brain, and cardioprotective activities, RS has attracted considerable attention [30,31,32,33]. However, its low bioavailability, poor solubility in water (0.021–0.030 mg/mL), short biological half-life, and chemical instability are the main challenges when exploiting its biological activity. In addition to that, the consumption of RS by oral dosing is limited due to the first-pass metabolism in the liver in humans [34,35]. To overcome such drawbacks, encapsulation of RS into a polymer-based nanocarrier system can be used to improve its activity [30,36]. There are many studies, which are focusing on various applications of RS. In the study of Jayan et al. (2019), to enhance RS bioavailability, RS-loaded zein nanoparticles were developed by electrohydrodynamic atomization (EHDA). The zein nanoparticles within a size range of 230 nm to 330 nm displayed an improved release profile compared to their native form. It was claimed that the produced nano encapsulated particles could be potential ingredients for the food industry [33]. Zhang et al. (2019) investigated RS anchored star-shaped electrospun fibers composed of poly(ε-caprolactone)-carboxylic acid/poly(l-lactide) (PCL-COOH/PLLA) on neonatal human dermal fibroblasts (NHDF) for skin tissue engineering. These obtained fibers demonstrated no cytotoxic effect and facilitated the viability of NHDF cells [31].

A variety of techniques have been employed to produce nanoscale polymeric particles. Spray drying, coacervation/precipitation, solvent extraction/evaporation, ionic gelation are some examples for conventional methods [37,38]. Besides these nanoparticle fabrication techniques, the EHDA process, also called electrospray technique, is a convenient method due to its properties like the cost-effectiveness and short duration of operation, one-step easy production process and production of near-uniform sized particles by easily tailoring system parameters such as voltage, flow rate, collector distance and polymer concentration [9,33,39,40,41]. EHDA has been widely practiced in the pharmaceutical industry and biomedical applications over the past years [42,43]. The principle lying behind EHDA is the atomization of a conductive solution under the influence of an electric field [44,45]. When the surface tension stress of charged solution is overcome by electrical forces, the charged solution jet breaks down into very fine particles via Coulomb repulsion forces [40,45,46]. Then, nanoparticles are formed after the evaporation of the solvent [47].

When the crucial advances of nanostructured systems within various biomedical applications and the significant outcomes obtained from previous research on levan as a biopolymer are considered, levan-based nanosized systems hold great potential and have been previously studied for different applications [48]. Encapsulation of bovine serum albumin into levan nanoparticles created by self-stacking nanoprecipitation method [28], encapsulation of indocyanine green into self-assembled levan nanoparticles for targeted breast cancer imaging [49], production of levan coated silver nanoparticles showing bactericidal effect [50] are some examples for previously studied levan-based nanocarrier systems produced by different methods. However, systematic studies are still needed based on levan nanoparticles to enhance their use as carriers for drugs or active molecules. On the other hand, EHDA has been shown as a simple, versatile, and scalable technique for fabricating functional nanosized polymeric particles [47]. To the best of our knowledge, levan-based nanostructures produced via EHDA technique is limited to only one study where a commercial levan polymer produced with *Zymomonas mobilis* (Real Biotech Co, Ltd., Korea) [16,51] was used for the formation of amphiphilic levan-clasped self-assembly encapsulating hydrophobic magnetic nanoparticles (Amp-SA-M) via co-electrospraying for in vivo imaging [52]. On the other hand, there is no study about levan-based nanoparticles produced via EHDA that aimed to develop a delivery system for drugs or bioactive molecules. Hence, this study aimed to be the first systematic investigation of RS-loaded hHL nanoparticles fabricated via EHDA. For this, firstly, all polymer solutions were physically characterized in terms of density, electrical conductivity and surface tension. Then, hHL-based nanoparticles were obtained by optimizing parameters of the EHDA process and then characterized for their morphological, chemical and biological properties. Additionally, RS concentration on encapsulation efficiency and in vitro release profiles of the nanoparticles were evaluated. Results showed that the fabricated RS-loaded hHL nanoparticles may have potential use in drug delivery systems for wound healing and tissue engineering applications.

## 2. Materials and Methods

### 2.1. Materials

*Halomonas* levan (HL) was produced by recombinant *H. smyrnensis* levansucrase enzyme (EC 2.4.1.10) and purified as reported previously by Kirtel et al. (2018) [18]. Dimethyl sulfoxide (DMSO) was purchased from Merck, Darmstadt, Germany. Resveratrol (RS) was obtained from Sigma-Aldrich (R5010), St. Louis, MO, USA. Human dermal fibroblast cell lines (PCS-201-012) were obtained from Prof. Betül Yılmaz (Marmara University, Istanbul, Turkey).

### 2.2. Enzymatic Production and Purification of Levan

Levan production was performed with levansucrase enzyme in a 2 L substrate solution containing K_2_HPO_4_ (5.36 g/L), KH_2_PO_4_ (2.62 g/L), commercial sucrose (100 g/L), Çamaltı salt (204.5 g/L), and ethylenediaminetetraacetic acid (EDTA) (15 mM). After preparing the substrate solution, it was filtered through with a technical filter paper to seperate undissolved impurities from salt and the pH value was set to 5.85–5.90. The purified levansucrase enzyme was added to the substrate solution and stirred. Then, it was left for incubation at 15 °C overnight without agitation. The next day, ethanol precipitation was applied by adding 3 L of 96% ethanol into the levan solution and left overnight at −20 °C. Later, the clear supernatant was separated, and the rest was centrifuged at 8000× *g* for 20 min. The pellet was dissolved with distilled water and then dialyzed via 13 kDa cut-off size dialysis tubing against tap water. The dialysis continued for six days by changing water two times a day. For the last three days, the water was switched to distilled water and the procedure was repeated until the end. The levan solution was subjected to weak-anion exchange chromatography to purify the polymer. The purified levan was dried by an air drier at 55 °C. Then, the dried product was collected in the desiccator before milling. Levan in powder form was obtained after milling and stored in the refrigerator or freezer.

### 2.3. Hydrolysis of Levan

Levan was hydrolyzed by microwave-assisted acid hydrolysis. To a solution of levan (10 gr) in distilled water (171.5 mL), acetic acid (3.5 mL) was added. After 10 min of mixing, the solution was exposed to a microwave for 60 s operating at 60% of its total power. To stop hydrolysis, the solution was kept at 4 °C for overnight. The hydrolyzed polymer was precipitated with ethanol by storing it at −20 °C overnight and then it was centrifuged at 8000× *g* for 20 min. The pelleted polymer was dried in a vacuum oven for 24 h at 46 °C. The hydrolyzed levan (hHL) was obtained in powder form after grinding. The product was characterized by FTIR and GPC analysis. The molecular weights of HL and hHL were determined as 51,300 ± 0.262 kDa and 21,330 ± 0.524 kDa, respectively, according to GPC analysis. 

### 2.4. Preparation and Characterization of Polymer Solutions

hHL levan was dissolved in 10 mL of DMSO at the concentration of 5% (*w*/*v*) with magnetic stirring (Wise Stir^®^, MSH-20 A, Wertheim, Germany). After polymer was completely dissolved, RS was dissolved in 5% (*w*/*v*) hHL solution at the concentrations of 0.05% and 0.1% (*w*/*v*). The density of the solution was measured with a standard density bottle (10 mL). A surface force tensiometer (Sigma 703D, Attension, Darmstadt, Germany) with a platinum ring was used to characterize the surface tension of the solution. The viscosity of solutions was determined using a digital viscometer (DV-E, Brookfield AMETEK, Middleborough, MA, USA). All the measurements were carried out at room temperature (25 °C).

### 2.5. Experimental Set-Up of EHDA and Fabrication of Levan Nanoparticles

**Figure 1:**
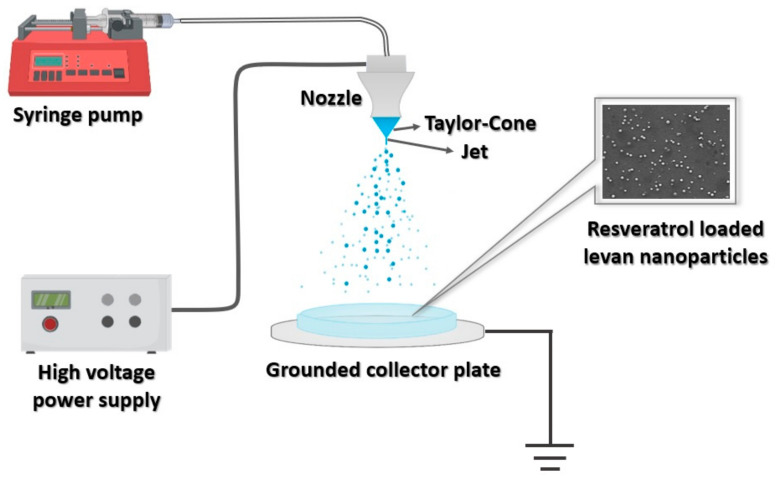
Schematic illustration of the experimental set-up of the EHDA process for the fabrication of nanoparticles.

To fabricate nanoparticles, a Basic System (Inovenso, Istanbul, Turkey) EHDA equipment was used. The EHDA set-up, which is illustrated in Figure 1, is composed of a high-voltage power supply, a syringe pump containing the spray solution (IPS-12, Inovenso, Istanbul, Turkey), a grounded aluminium collector plate, and a metal capillary (0.51 mm of internal diameter and 0.82 mm of external diameter). During the operation of the system, the hHL solution was pumped throughout the pipe to the metallic needle. The solution was electrosprayed onto a glass Petri dish with a flow rate ranging from 0.8 to 8 µL/min and the voltage was set up between 16 kV and 25 kV. The distance between the collector and the needle tip was set to 10 cm and 15 cm. The nanoparticles were kept in a desiccator overnight and then stored in a freezer until further use.

### 2.6. Morphological and Chemical Characterization of Nanoparticles

#### 2.6.1. Scanning Electron Microscopy (SEM) and Optical Microscopy

The morphology of levan nanoparticles was characterized by a scanning electron microscope (SEM, EVO LS 10, ZEISS). All nanoparticles obtained on glass slides were dried in an oven at 37 °C for about 24 h. The SEM samples were pre-coated with Au-Pd using a Quorum SC7620 Mini Sputter Coater for 120 s. The accelerating voltages during scanning were 5 kV and 10 kV. The average particle diameter of the nanoparticles was examined via an optical microscope (Olympus AnalySIS, Tokyo, Japan).

#### 2.6.2. Fourier-Transformed Infrared (FTIR) Spectroscopy

Characterization of the functional groups and bonding interactions of the hHL, RS, and RS-loaded hHL nanoparticles were performed by Fourier-transformed infrared (FTIR) spectroscopy (Jasco FT/IR-4700, Tokyo, Japan) analysis. The measurements were recorded at the mid-IR region from 4000 to 400 cm^−1^ at 4 cm^−1^ resolution under ambient temperature (25 °C).

#### 2.6.3. X-ray Diffraction (XRD)

X-ray diffraction (XRD) analysis was performed to observe the crystal structure of the produced hHL nanoparticles. It was operated with a Cu source (λ = 1.54060 A°) at a voltage of 40 kV, current of 30 mA, the scan range of 10–80°, the scan speed of 2 (°/min) and the preset time of 0.60 s.

### 2.7. Encapsulation Efficiency and In Vitro Release Study

The RS-loaded hHL nanoparticles (5 mg) were dissolved in 10 mL of DMSO for at least 1 h under vigorous stirring. Then, the amount of the drug was calculated by measuring the solution via UV- visible spectrophotometer (UV Mini 1280, Shimadzu, Japan) at 309 nm. The RS concentration was determined using the standard calibration curve obtained from absorption values of 2.5–20 µg/mL RS with an R^2^ value of 0.996. The % encapsulation efficiency was evaluated using the following equation:

(1) $$\mathrm{EE}\;\left(\%\right)=\frac{\mathrm{Amount}\;\mathrm{of}\;\mathrm{drug}\;\mathrm{in}\;\mathrm{nanoparticles}}{\mathrm{Total}\;\mathrm{amount}\;\mathrm{of}\;\mathrm{added}\;\mathrm{RS}\;\mathrm{in}\;\mathrm{nanoparticles}}\;\times \;100\;$$

The in vitro release study of RS-loaded nanoparticles was performed in pH 7.4 and pH 5.0 Phosphate Buffer Saline (PBS). 5 mg of RS-loaded nanoparticles were immersed in 1 mL of PBS in a thermal shaker (BIOSAN TS-100, Riga, Latvia), constantly stirring at 0.1512× *g* and 37 °C. 1 mL of release medium was withdrawn at determined time intervals throughout 24 h and measured at 309 nm by UV spectrophotometer. Then, 1 mL of fresh PBS solution was added again to continue the release study. All the release tests were conducted in triplicate.

### 2.8. In Vitro Drug Release Kinetics

The drug release mechanisms and release profiles from nanoparticles were examined and evaluated via kinetic models (Zero-order, First-order, Higuchi, and Korsmeyer–Peppas) as represented in the below equations [53,54].

(2) $$\mathrm{Zero}-\mathrm{order}:\;{Q=Q}_{0}{+k}_{0}t\;$$

(3) $$\mathrm{First}-\mathrm{order}:\;{\log\;Q}_{t}{=\log\;Q}_{0}-k_{1}t/2.303\;$$

(4) $$\mathrm{Higuchi}:\;{Q=k}_{H}t^{1/2}$$

(5) $$\mathrm{Korsmeyer}\text{–}\mathrm{Peppas}:\;{Q=\mathrm{kt}}^{n}$$

Here, Q is the amount of cumulative released drug at time t; k_0_, k_1_, k_H_, and k are the kinetic constants of respective above-mentioned equations; n is the release exponent of the Korsmeyer–Peppas model.

### 2.9. In Vitro Biocompatibility Assay

hHL and RS-loaded hHL electrosprayed particles (hHL/0.05RS and hHL/0.1RS) sprayed onto round glass slides, were tested to assess biocompatibility with human dermal fibroblast cell lines (PCS-201-012) via WST-1 (4-[3-(4-iodophenyl)-2-(4-nitrophenyl)-2H-5-tetrazolio]-1,3-benzenedisulfonate) (Roche Applied Science, Mannheim, Germany) cell proliferation and viability assay. For this, surfaces were presterilized by 2% penicillin-streptomycin in PBS for 30 min and were suspended in DMEM. After the sterilization process, sprayed surfaces were placed in 48 well plates, and dermal fibroblast cells at the 70% confluency were seeded onto samples at the density of 5 × 10^4^ cell/well and incubated for 24 h at 37 °C in humidified air containing 5% CO_2_. At the end of the incubation period, WST-1 reagent was added onto wells and incubated for 2 h in the dark at 37 °C in 5% CO_2,_ and absorbance was measured at 405 nm by Biotek Cytation 3 (Winooski, VT, USA). Untreated cells (cells on wells without any sample) were used as control and considered 100% viable. 

#### Cell Behavior on Electrosprayed Particle Surfaces

Growth and attachment of PCS-201-012 cells on electrosprayed surfaces were further investigated with fluorescence microscopy and SEM images. For this, surfaces were incubated with cells for 24 h and fixed with 4% paraformaldehyde (PFA) in PBS solution and washed with 1× PBS for two times. For fluorescence microscopy, nuclei of the cells were stained with DAPI (4′,6-diamidino-2-phenylindole dihydrochloride, AppliChem, Darmstadt, Germany) in the dark. After fixation and dying (surface for fluorescence microscopy), all samples were dehydrated via rinsing in increasing ethanol concentrations (70%, 80%, 90%, and 100%) and air dried in the dark before further investigations with SEM and fluorescence microscopy. 

### 2.10. Statistical Analysis

Statistical analysis of viability results was performed with Graph Pad V 5.0 prims analysis program and One-Way ANOVA followed by post hoc Tukey was performed for in vitro studies. All experiments were performed in triplicate and data were presented as mean and 95% confidence interval (CI). Differences were considered significant if *p* < 0.05.

## 3. Results and Discussion

### 3.1. Physical Characterization of Polymer Solutions

Various parameters are playing crucial roles in the EHDA process [44,45]. Hence, the density, electrical conductivity, surface tension, and viscosity that show characteristic properties of all solutions were investigated, and the results are demonstrated in Table 1. The addition of RS into the polymer solution increased the surface tension of the polymer solution only very slightly. When comparing both hHL solutions containing RS, the density and electrical conductivity increased but the viscosity decreased with increasing RS amount as seen in Table 1.

### 3.2. Production of Nanoparticles

Along with the physical properties of polymer solutions, the EHDA process is also affected by the applied voltage, flow rate, the distance between collector plate and nozzle, and solvent type [55]. In preliminary experiments, hHL dissolved in water (5% *w*/*v*) could not be appropriately electrosprayed which might be caused by the electrical breakdown in the air due to the high surface tension of water as also reported in the literature [44]. Therefore, DMSO, in which both RS and levan polymer can be dissolved, was chosen as solvent. Furthermore, a stable Taylor cone-jet and better nanoparticles in terms of shape and size were obtained by applying a voltage at the values of 18 kV and 20.5 kV. Additionally, the nanoparticles with smaller sizes were achieved by decreasing the flow rate and increasing the collector distance to 15 cm. 

### 3.3. Morphological Analysis of Produced Nanoparticles

**Figure 2:**
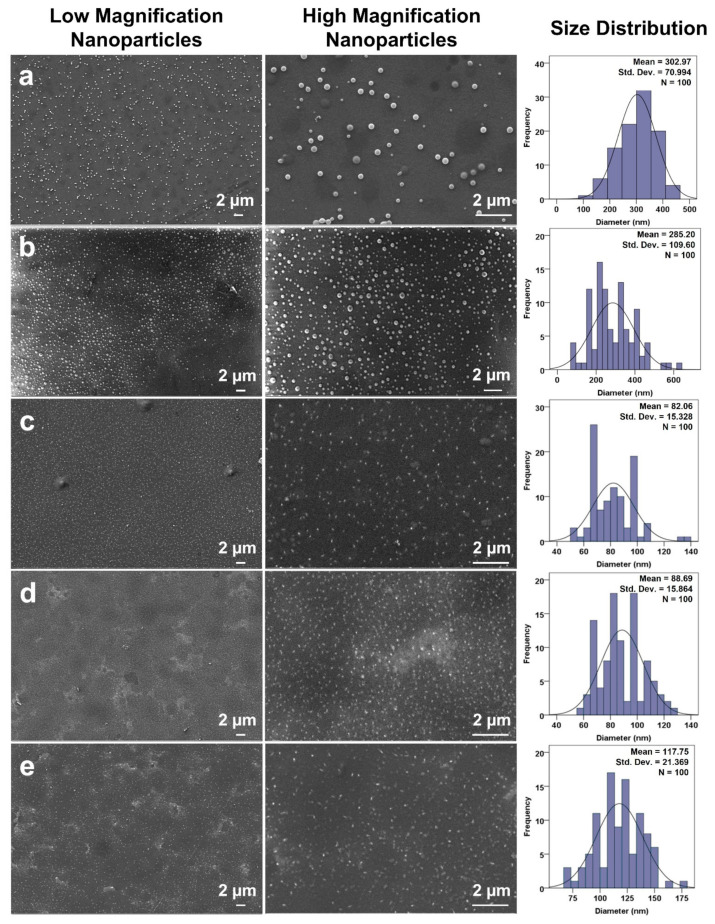
SEM micrographs of prepared nanoparticles at different production conditions and their size distributions. hHL solution was sprayed on (**a**) 8 µL/min, 18 kV and 10 cm distance, (**b**) 2.5 µL/min, 18 kV, and 10 cm distance, (**c**) 2.5 µL/min, 20.5 kV, and 15 cm. (**d**) hHL/0.05RS solution, (**e**) hHL/0.1RS were sprayed on 2.5 µL/min, 20.5 kV, and 15 cm.

SEM micrographs of hHL and RS-loaded hHL nanoparticles collected and their size distributions are shown in Figure 2. As seen in the figure, the particles produced have a smooth surface and are spherical in shape. The mean particle diameter was found as 302.97 ± 70.99 nm under the conditions of flow rate as 8 µL/min, applied voltage as 18 kV, and distance as 10 cm. Decreasing the flow rate value to 2.5 µL/min at constant electrical power and collector distance developed smaller nanoparticles with an average diameter of 285.20 ± 109.60 nm as shown in Figure 2a,b. Thus, the flow rate is a crucial parameter affecting the size of the particles and smaller particles are obtained as flow rate decreases [45,55]. During optimization of particle collection, change in the distance from 10 cm to 15 cm within application voltage at 20.5 kV enhanced drying of particles and allowed to obtain much smaller particles than previously fabricated ones. In this way, the EHDA of % 5 *w/v* hHL solution was optimized at 2.5 µL/min flow rate, 20.5 kV voltage, and 15 cm collector distance. The mean particle diameter of hHL nanoparticles without RS was found as 82.06 ± 15.33 nm as seen in Figure 2c. RS-loaded hHL nanoparticles had an average particle diameter of 88.69 ± 15.86 nm and 117.75 ± 21.37 nm as observed in Figure 2d,e, respectively. The mean particle size of RS-loaded hHL nanoparticles was slightly larger than the hHL particles. When comparing the obtained particle sizes, a slight increase in particle diameter was observed as the RS concentration increased. Therefore, the size and morphology of the particles were optimized by controlling the EHDA parameters. SEM micrographs in Figure 2c–e showed that fabricated hHL and RS-loaded hHL nanoparticles not only had a spherical form but were also distributed as monodisperse in size. RS was successfully loaded into particles within the optimized system parameters. There was no formation of crystals or clumps resulting from the loading and consequent dispersion of the active molecule as reported in literature with different studies [38,56,57].

### 3.4. Fourier Transform Infrared Spectroscopy (FTIR)

**Figure 3:**
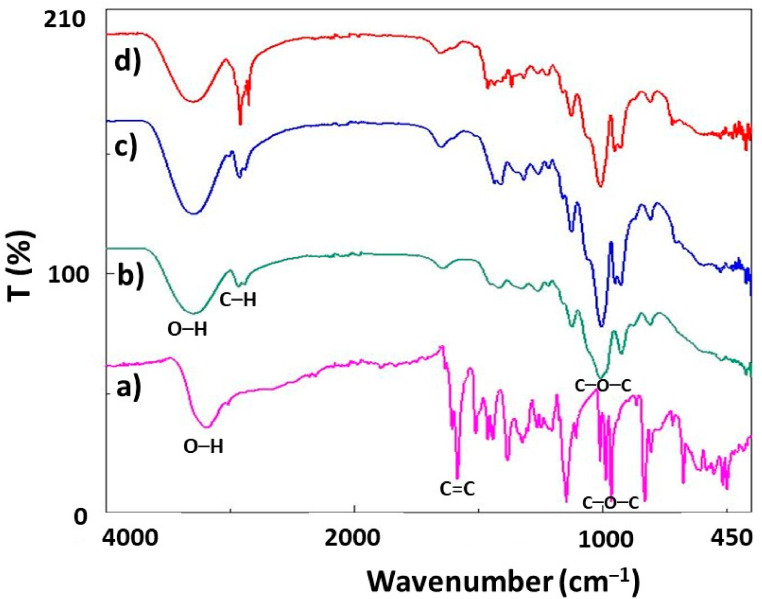
FTIR spectrums of the (**a**) RS, (**b**) hHL, (**c**) hHL/0.05RS, (**d**) hHL/0.1RS specimens.

FTIR analysis was performed to examine the functional groups of the prepared specimens. Molecular structures of RS, hHL, and the FTIR spectra of nanoparticles produced at different concentrations are shown in Figure 3. In Figure 3a, O–H stretching at 3195 cm^−1^ was observed in the absorption spectra of pure RS due to the alcoholic group [58]. The stretching related to aromatic double bonds (C=C bonds) was observed in bands at 1611 cm^−1^ [59]. Additionally, C–C olefinic stretching at 1583 cm^−1^ and the peak at 965 cm^−1^ indicated C–O–C stretch in the transform of RS [58]. For hHL (Figure 3b), the characteristic absorption band at 3301 cm^−1^ is because of the OH stretching of fructofuranose rings [25]. 2933 cm^−1^ are due to the carbon–hydrogen (C–H) stretching vibration of fructose residues [27]. Additionally, three bands at 1415, 1008, 921 cm^−1^ corresponding to C–O–C were observed [20]. The characteristic peaks of RS were obtained only weakly in the spectra for the drug-loaded nanoparticles due to the low amounts.

### 3.5. XRD Analysis

**Figure 4:**
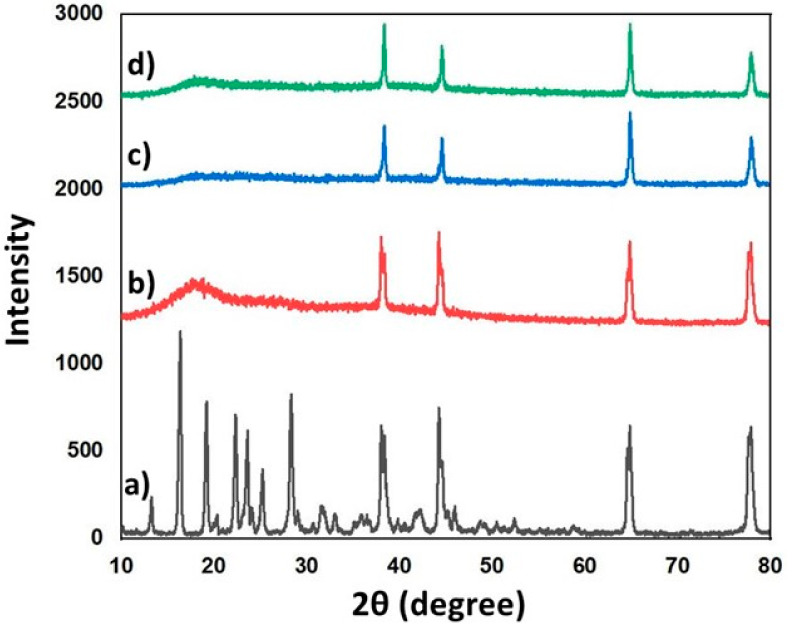
XRD spectra of (**a**) RS, (**b**) hHL, (**c**) hHL/0.05RS, (**d**) hHL/0.1RS specimens.

X-ray diffraction results of RS, hHL, hHL/0.05RS, and hHL/0.1RS samples were shown in Figure 4. The XRD spectrum of RS exhibited sharp peaks at 2θ of 13.26°, 16.4°, 19.2°, 22.32°, 23.62°, 24.26°, 28.34°, 38.04°, 44.22°, 64.84°, 77.88°, which confirmed the crystalline nature of the drug (Figure 4a) [60,61]. These characteristic peaks were also observed in the diffraction pattern of RS-loaded hHL samples though their intensities were weaker because of the relatively low RS content in the mixture. Diffraction peaks at 17.66°, 38.06°, 44.26°, 64.84° and 77.92° 2θ were observed for hHL (Figure 4b) [62]. In RS-loaded hHL nanoparticles, it is confirmed that RS was encapsulated in the hHL nanoparticles in its crystalline form (Figure 4c,d). A fall in the intensity of diffraction peaks was observed with the inclusion of RS in hHL solution. It reduced the diffraction intensity, but no shift in angle values was observed. This indicated that RS was effectively dispersed in the nanoparticle.

### 3.6. Encapsulation Efficiency

The encapsulation efficiencies of hHL/0.05RS and hHL/0.1RS nanoparticles were calculated as 13.8 ± 1.3% and 6.54 ± 0.04%, respectively. Hence, the encapsulation efficiency was found to be affected by the amount of RS loaded such that when the amount of loaded RS was increased two-fold in the formulation, the encapsulation efficiency was reduced to almost half. This might have resulted from complete entrapment of RS occurring during the flight time and the polymer may not be able to encapsulate the active material sufficiently as also reported in the studies of Jayan et al. (2019) and Bhushani et al. (2017) [33,57].

### 3.7. In Vitro Release Study

**Figure 5:**
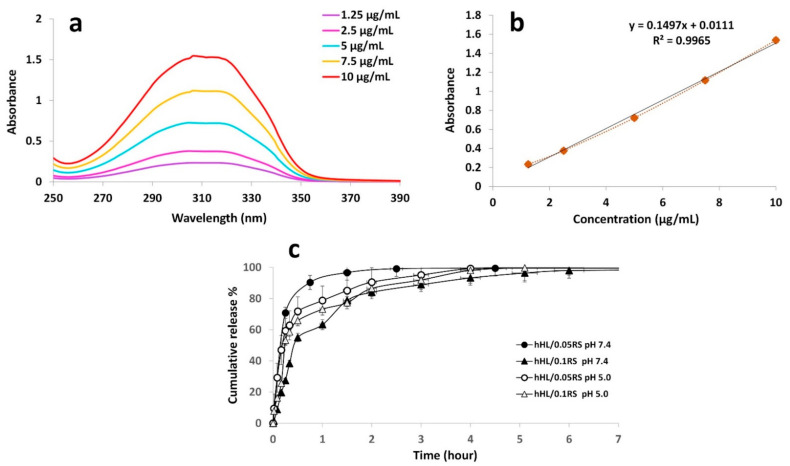
(**a**) UV absorbance spectra of RS and (**b**) calibration curve of RS at 309 nm, and (**c**) in vitro release studies of RS from nanoparticles at pH 7.4 and pH 5.0.

The release behaviors of RS-loaded hHL nanoparticles assessed in different pH media were shown in Figure 5c. Firstly, the concentration range of RS from 1.25 to 10 µg/mL was used to plot the UV absorbance spectra shown in Figure 5a. Then, a linear standard calibration curve was constructed by plotting the graph of the absorbance at 309 nm of RS against concentration (R^2^ = 0.99) for the quantitative determination of release data (Figure 5b). The release of RS from nanoparticles was carried out in PBS pH 7.4 to mimic the physiological conditions in the body [33], and pH 5.0 to mimic the natural environment of skin and wound [53,63,64] at a controlled temperature of 37 °C. In Figure 5c), a burst effect was observed in all formulations. At physiological pH, hHL/0.05RS nanoparticles demonstrated rapid release from the particles around 70% within the first 15 min, and then most of the rest were released within 2.5 h. On the other hand, initial burst release from hHL/0.1RS (pH 7.4) was observed, and approximately 63% of the active molecule was released during the first hour, while hHL/0.05RS nanoparticles already released more than 90% at that time. High amounts of RS were released from hHL/0.1RS particles within the first 6 h with negligible release afterwards up to 24 h. At a more acidic pH environment, hHL/0.05RS released 59% of the active molecule while hHL/0.1RS released 53% of the active molecule within 15 min, and both samples continued to release RS about 5 and 6 h, respectively. Therefore, hHL/0.1RS at pH 7.4 showed the most gradual release profile compared to other samples. The obtained results indicate that RS concentration in the formulation played a significant role in the release profiles of the samples, and the release slowed down as the amount of RS increases. As similarly mentioned in the literature, the burst effect was associated with the ratio of RS in the formulation and the amount of RS near the particle surface. A rapid release occurred when the RS was likely to be weakly bound or closer to the particle surface compared to the content of the particle core. Increasing the loading of the active molecule increases particle size. This allows control in burst release and to obtain a slower release profile [11,33].

A variety of kinetic models were used to figure out the release kinetics of RS-loaded nanoparticles. The zero-order kinetics define processes based on a constant drug release from the delivery system as being independent of drug concentration. Unlike the zero-order, the release rate of the drug is dependent on the drug concentration according to the first-order model. Besides these, drug release from a matrix system controlled by diffusion is described by the Higuchi model. The Korsmeyer–Peppas model explains the type of diffusion followed by drugs released from a polymeric system [54,65]. As shown in Table 2, the correlation coefficients (R^2^) and the kinetic constants were obtained by applying the release data from the samples to the kinetic models such as zero-order, first-order, Higuchi, and Korsmeyer–Peppas. The regression coefficient values were used to find the most suitable release model for this study. Based on R^2^ values, samples did not follow the Korsmeyer–Peppas model as they had the lowest value of R^2^ as shown in the table. In accordance with obtained data from kinetic analysis, the kinetic model which the release data were best fitted with was first-order release model which has the highest values of R^2^ for all samples. Therefore, the released amount of RS displays a decreasing tendency as a function of time [65].

### 3.8. In Vitro Biocompatibility Assay

**Figure 6:**
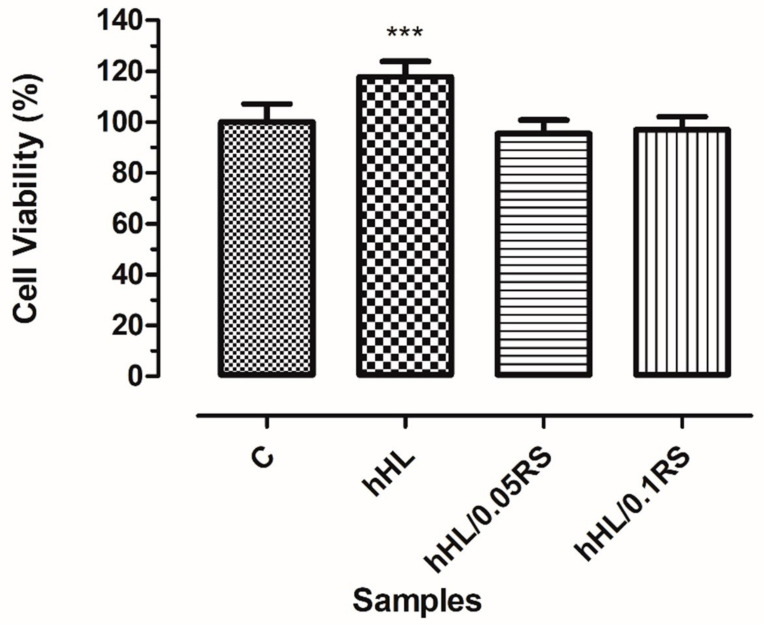
Cell viability results of PCS-201-012 cells after being cultivated with electrosprayed surfaces for 24 h. (A *p* value below 0.05 is represented as; 0.001 to 0.01 as ***).

Proliferation and cell viability of PCS-201-012 cells on electrosprayed surfaces were investigated for 24 h, whereas the viability of control, hHL, hHL/0.05RS, and hHL0.1RS samples were observed as 100 ± 15.30, 117.7 ± 14.48, 95.50 ± 10.57, and 97 ± 11.21%, respectively. hHL coated surfaces showed the highest viability, while samples with increasing RS did not show any significancy on cell viability when compared with each other (Figure 6).

**Figure 7:**
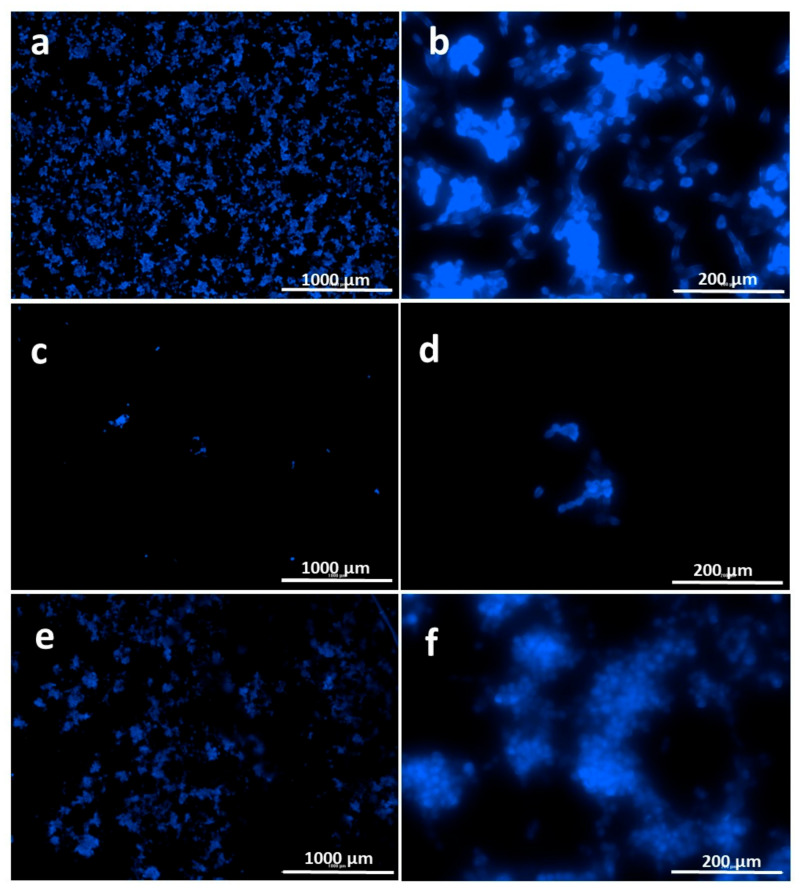
Cell attachment images of DAPI stained PCS-201-012 cells after being cultivated with electrosprayed surfaces with different magnifications, (**a**,**b**) hHL, (**c**,**d**) hHL/0.05RS, and (**e**,**f**) hHL/0.1RS for 24 h.

**Figure 8:**
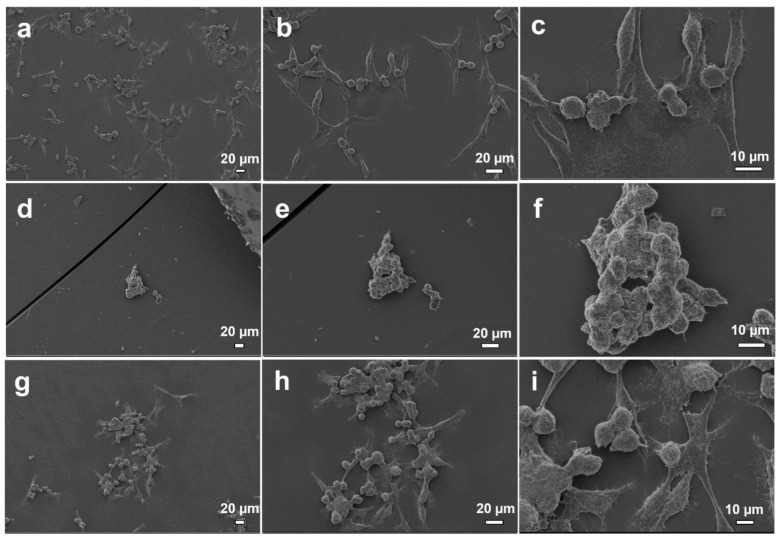
SEM images of PCS-201-012 cells adhesion after being cultivated with electrosprayed surfaces with different magnifications, (**a**–**c**) hHL, (**d**–**f**) hHL/0.05RS, (**g**–**i**) hHL/0.1RS.

Cell behavior on electrosprayed surfaces was observed via fluorescent microscopy (Biotek cytation 3, Winooski, VT, USA). DAPI stained cell nuclei after 24 h incubation with surfaces were shown in Figure 7, and SEM images of cell adhesion were shown in Figure 8. It was observed from viability graph, SEM and fluorescence images that surfaces were biocompatible and cells attached on surfaces sprayed with hHL and attached cell population increased with the increase in RS content of particles (Figure 7 and Figure 8).

Biocompatibility, anticoagulant activity, anticancer activity, and cell proliferating abilities are reported for HL levan and chemically modified derivatives previously [19,24,25,26,29,66,67,68,69]. Demirci et al. (2020) investigated the biocompatibility of amphotericin B-loaded HL hydrogels with murine fibroblast cell line (L929) and observed 93% viability [70]. Enhanced cell proliferation and collagen expression are reported previously for injectable levan-pluronic acid and carboxymethyl cellulose (CMC) hydrogels by Choi et al. (2018) [71]. Cansever Mutlu et al. (2021) investigated the anticancer activity of paclitaxel-loaded quaternized HL coated lipidic nanoparticles (208 nm) on human lung A549 cancer cells, and high cytotoxicity was observed [72]. Suktham et al. (2018) investigated RS-loaded silk fibroin nanoparticles (200–400 nm) and reported strong inhibition on the growth of human colorectal cancer cell line (Caco-2), while no cytotoxicity was observed for skin fibroblasts (CRL 25-22) [73]. Hyaluronic acid-ceramide and soluplus based electrosprayed nanocomposites were proposed for tumor-targeted RS release by Lee et al. (2016) on CD44 receptor expressed MDA MB 231 human breast cancer cells [74]. Cell viability below 30% is considered as cytotoxic according to ISO standard 10, 993-5. Results obtained from the electrosprayed surfaces of this study have shown the bioactivity of levan and improved efficiency in RS activity once its encapsulated. It can be concluded that electrosprayed particles are safe for healthy cells and can be used as drug delivery systems. 

It is clearly shown from Figure 6 that hHL samples improved cellular viability and cells attached on those surfaces. Biocompatibility of the polymers are known to be affected by their properties such as molecular weight, charge, structure (linearity or branched), flexibility and hydrophilicity and branched molecules are known to neutralize surface charge of the cells [75]. Cells are known to prefer surfaces to attach and proliferate that have hydroxyl (CH2OH) or carboxylic acid (-COOH) groups [76]. The branching degree of the polysaccharides is a structural feature associated with the presence of linked monosaccharide residues or linked chains to their backbone. Depending on this structural feature, solubility and other structural features will be affected, namely molecular weight and conformation. Partially branched hHL produced by *Halomonas* levansucrase enzyme with a higher number of free hydroxyl groups might affect fibroblast cell attachment and viability through increased hydrophilicity on surface. Thus, improved cellular interaction could be expected on surfaces coated with these techniques. 

## 4. Conclusions

In this study, the hydrolyzed form of levan polysaccharide produced by recombinant *H. smyrnensis* levansucrase enzyme (EC 2.4.1.10) was used to develop nanocarriers with EHDA technique and they were investigated their potential use for controlled drug release by using RS as a model drug. It has been demonstrated that hHL-based nanoparticles can be used to encapsulate a bioactive molecule such as RS. The fabrication conditions of the nanoparticles were optimized at 2.5 µL/min flow rate, 20.5 kV voltage, and 15 cm collector distance. The particles displayed smooth surfaces, were spherical in shape and they could be obtained in smaller sizes after optimization of EHDA parameters. The particle size diameters were reduced to 82.06 ± 15.33 nm for hHL nanoparticles from 302.97 ± 70.99 nm. Additionally, the loading of RS into the particles slightly increased the particle size. The loading of RS was achieved under these optimized conditions and neither drug crystals nor clusters were observed. In release studies, more than 90% of RS was released from hHL/0.05RS nanoparticles within 2.5 h at pH 7.4. However, hHL/0.1RS particles completed its release at pH 7.4 for about 24 h by releasing high amounts of RS during the first 6 h and then following a negligible release. Under more acidic conditions (pH 5.0), the release of RS from hHL/0.05RS and hHL/0.1RS were completed around 5 and 6 h, respectively. Compared to the profile of hHL/0.05RS nanoparticles at both pH conditions, a more gradual release profile was observed in hHL/0.1RS samples. hHL/0.1RS exhibited the slowest release profile compared to others. All samples followed the first-order release mechanism. In the light of in vitro cell viability, fluorescent microscopy, and SEM images of cell adhesion results, electrosprayed nanoparticles were found to be biocompatible. In conclusion, hHL-based nanoparticles obtained via the EHDA technique may have potential use in drug delivery systems for wound healing and tissue engineering applications and it offers enhancement in cellular interactions for surfaces that could be coated with these eletrosprayed particles.

## Figures and Tables

**Table 1:** Physical characteristics of solutions used in EHDA process.

Solutions	Density (g/mL)	Electrical Conductivity (µS/cm)	Surface Tension (mN/m)	Viscosity (mPA s)
hHL	1.131 ± 0.001	40.6 ± 0.2	42.70 ± 0.07	634.7 ± 0.5
hHL/0.05RS	1.085 ± 0.005	39.5 ± 0.1	43.40 ± 0.08	658.7 ± 1.2
hHL/0.1RS	1.095 ± 0.001	47.6 ± 0.2	43.60 ± 0.10	609.0 ± 2.6

**Table 2:** The release kinetics of RS-loaded nanoparticles.

	Zero Order		First Order		Higuchi		Korsmeyer–Peppas	
Samples	R^2^	k_0_	R^2^	k_1_	R^2^	k_H_	R^2^	n
hHL/0.05RS (pH 7.4)	0.193	2.501	0.794	−0.186	0.403	15.589	0.082	0.325
hHL/0.1RS (pH 7.4)	0.551	8.854	0.895	−0.196	0.820	34.707	0.494	0.5949
hHL/0.05RS (pH 5.0)	0.603	19.628	0.958	−0.454	0.832	47.043	0.119	0.289
hHL/0.1RS (pH 5.0)	0.651	16.775	0.962	−0.431	0.855	44.198	0.224	0.381
